# Experiment and Artificial Neural Network Prediction of Thermal Conductivity and Viscosity for Alumina-Water Nanofluids

**DOI:** 10.3390/ma10050552

**Published:** 2017-05-19

**Authors:** Ningbo Zhao, Zhiming Li

**Affiliations:** College of Power and Energy Engineering, Harbin Engineering University, Harbin 150001, China; zhaoningbo314@126.com

**Keywords:** nanofluids, thermal conductivity, viscosity, artificial neural network, experimental data

## Abstract

To effectively predict the thermal conductivity and viscosity of alumina (Al_2_O_3_)-water nanofluids, an artificial neural network (ANN) approach was investigated in the present study. Firstly, using a two-step method, four Al_2_O_3_-water nanofluids were prepared respectively by dispersing different volume fractions (1.31%, 2.72%, 4.25%, and 5.92%) of nanoparticles with the average diameter of 30 nm. On this basis, the thermal conductivity and viscosity of the above nanofluids were analyzed experimentally under various temperatures ranging from 296 to 313 K. Then a radial basis function (RBF) neural network was constructed to predict the thermal conductivity and viscosity of Al_2_O_3_-water nanofluids as a function of nanoparticle volume fraction and temperature. The experimental results showed that both nanoparticle volume fraction and temperature could enhance the thermal conductivity of Al_2_O_3_-water nanofluids. However, the viscosity only depended strongly on Al_2_O_3_ nanoparticle volume fraction and was increased slightly by changing temperature. In addition, the comparative analysis revealed that the RBF neural network had an excellent ability to predict the thermal conductivity and viscosity of Al_2_O_3_-water nanofluids with the mean absolute percent errors of 0.5177% and 0.5618%, respectively. This demonstrated that the ANN provided an effective way to predict the thermophysical properties of nanofluids with limited experimental data.

## 1. Introduction

Considering the higher thermal conductivity of many solid materials, including Cu, CuO, TiO_2_, ZnO, Fe_3_O_4_, MgO, Al_2_O_3_ and graphite, dispersing solid particles in the conventional coolants (such as water, oil, ethylene glycol, refrigerant, etc.) is one of the most efficient ways to enhance the heat transfer process [1]. However, a large number of experimental results indicated that the lower suspension stability of large particles seriously limited the practical application of the traditional liquid-solid mixture. In the 1990s, the idea of nanofluids consisting of nanoparticles and base fluid was firstly introduced by Choi [2]. Due to the potential advantages in flow and heat transfer performance, nanofluids become a focus in the field of thermal science [3].

Thermal conductivity and viscosity are the most important physical parameters and play crucial roles for studying nanofluids. Over the last two decades, various experimental investigations have been published to evaluate the effects of nanoparticles on thermal conductivity and the viscosity characteristics of base fluids. References [4,5,6,7,8,9,10,11,12,13] respectively reviewed the experimental and theoretical developments of various nanofluids’ thermophysical parameters. According to their analysis, it could be found that the addition of nanoparticles did enhance the thermal conductivity and viscosity of base fluids in varying degrees. However, it was unfortunate that there were still many differences in the measurements of thermal conductivity and viscosity due to the effects of nanofluids manufacturing and measuring technologies [14]. In addition, considering the complex mechanisms, including nanoparticle heat transport [15,16], nano-shells at the interface between liquid and particle [17,18,19,20], Brownian motion [21,22], and clustering of nanoparticles [23,24], the thermal conductivity and viscosity of nanofluids are very difficult to predict accurately using the traditional model-based approach. For these cases, it is very valuable to further study the experimental characteristics and predictive modeling of nanofluids’ thermal conductivity and viscosity.

In recent years, with the development of artificial intelligence technology, various data-driven modeling approaches have been put forward to solve the thermal science problem [25,26]. Inspired by the biological brain, an artificial neural network (ANN) can effectively establish the relationship between the input and output variables without considering the detailed physical process, which attracts increasing attention in terms of predicting the thermophysical properties of nanofluids. Hojjat et al. [27] firstly analyzed the application of a three-layer feed forward neural network on the thermal conductivities prediction of various non-Newtonian nanofluids. They found that the ANN predicted values were in agreement with the experimental data. The average and maximum errors were only 1.6% and 5.8%, respectively. On this basis, Longo et al. [28], Mehrabi et al. [29,30], Ariana et al. [31], Esfe et al. [32,33,34,35,36,37,38], and Vakili et al. [39] successively designed different ANN models (such as a feed forward neural network, adaptive neuro-fuzzy inference system, diffusional neural networks, etc.) to further verify the effectiveness of ANN in the modeling and prediction of nanofluid thermal conductivity. All of their results demonstrated that ANN was an effective tool in comparison with the traditional model-based approach for describing the enhancement behavior of nanofluid thermal conductivity. In addition, attracted by the better nonlinear mapping and recognition abilities of ANN, Yousefi et al. [40], Mehrabi et al. [41], Zhao et al. [42,43], and Heidari et al. [44] also extended the ANN based modeling approach to the prediction of nanofluid viscosity. As reported in their analysis, ANN could be used for predicting the viscosity of nanofluids with satisfactory accuracy. 

Up to now, much valuable literature has demonstrated the effectiveness of different ANN models for modeling and predicting the thermalphysical properties of nanofluids. However, considering the data characteristics of nanofluids’ properties and the modeling process of an ANN, there are still many difficulties or obstacles to be resolved. For example, most of the developed ANN had only one output (thermal conductivity, viscosity, or density) and were usually trained by using a large number of samples, which were obtained from different experiments. Fewer publications discussed the multiple parameter modeling and prediction performance of ANNs, especially with limited experimental data. This may means that the application of ANNs in nanofluids is still in its infancy.

Based on the above background, this study presents a further investigation into the prediction of Al_2_O_3_-water nanofluid thermal conductivity and viscosity by using ANN and the limited experimental data. With the influences of nanoparticle volume fraction (1.31%, 2.72%, 4.25%, and 5.92%) and temperature (from 296 to 313 K), four different Al_2_O_3_-water nanofluids were prepared and measured. On this basis, a RBF neural network with multiple outputs was constructed and verified through the experimental data. 

## 2. Experimental Methods

### 2.1. Materials and Method of Preparing Nanofluids

In this study, a two-step method is used to manufacture the Al_2_O_3_-water nanofluids with different nanoparticle volume fraction. The spherical Al_2_O_3_ particles (Xuan Cheng Jing Rui New Material Co., Ltd, Xuancheng, China) with an average diameter of 30 nm, a purity of 99.9%, a density of 3.6 g/cm^3^, and the specific surface area of 15 m^2^/g are selected. During the manufacturing, the measurement of the masses of the nanoparticles is carried out by using an electronic balance with an accuracy of 1 mg. The volume fractions of the nanoparticles (1.31%, 2.72%, 4.25%, and 5.92%) are calculated according to the following function:

(1) $$\varphi_{p}=\frac{\rho_{f}\phi_{p}}{\rho_{p}+\rho_{f}\phi_{p}-\rho_{p}\phi_{p}}$$

where $\varphi_{p}$ is the volume fraction of the nanoparticle, $\phi_{p}$ is the weight fraction of the nanoparticle, and $\rho_{p}$ and $\rho_{f}$ are the densities of the nanoparticle and base fluid, respectively. 

To keep the stability of the nanofluids, the sodium dodecylbenzene sulfonate (SDBS) from Guangzhou Chemical Reagent Factory (Guangzhou, China) is added as the dispersing agent. The mass of the SDBS is quantitatively determined with the electronic balance and equal to that of nanoparticle, based on the existing experiment investigations [45,46]. Moreover, periodical magnetic stirring and ultrasonic oscillating are applied to prepare the nanofluids, considering that the fact that the stability process technologies of nanofluids in different studies are not very unified. The times of periodical magnetic stirring and ultrasonic oscillating are usually determined according to the actual conditions. In the present study, the corresponding times of the above stability process technologies are 12 h and 5 h, respectively.

**Figure 1:**
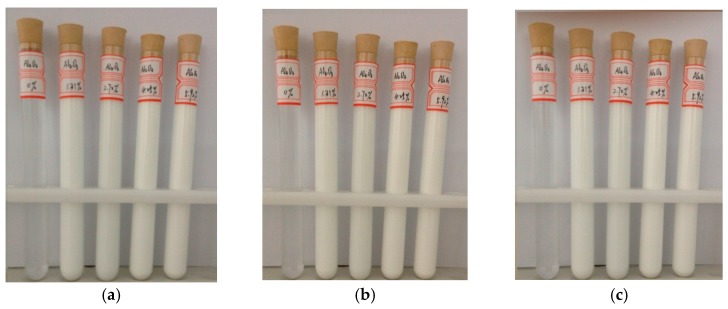
The manufactured Al_2_O_3_-water nanofluids with different nanoparticle volume fractions after different standing times; (**a**) 0 h; (**b**) 36 h; (**c**) 72 h.

**Figure 2:**
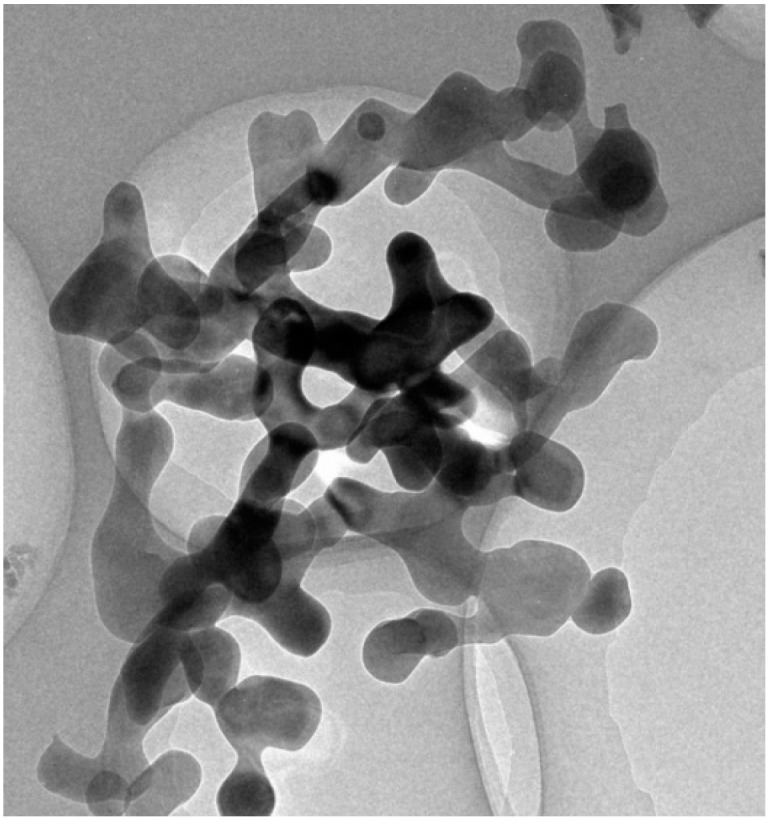
Scanning Electron Microscope (SEM) image of the Al_2_O_3_ nanoparticle with a volume fraction of 5.92% in water.

Figure 1 shows the manufactured Al_2_O_3_-water nanofluids after different standing times (0 h, 36 h, and 72 h). It can be seen that there is no obvious sedimentation for the above four different nanofluids, which means that they were manufactured successfully. In addition, Figure 2 presents the Scanning Electron Microscope (SEM) image of the Al_2_O_3_ nanoparticle with a volume fraction of 5.92% in water. It is clearly observed that most of the nanoparticles can be spherical and have good dispersion.

### 2.2. Method of Investigating the Thermal Conductivity and Viscosity

**Figure 3:**
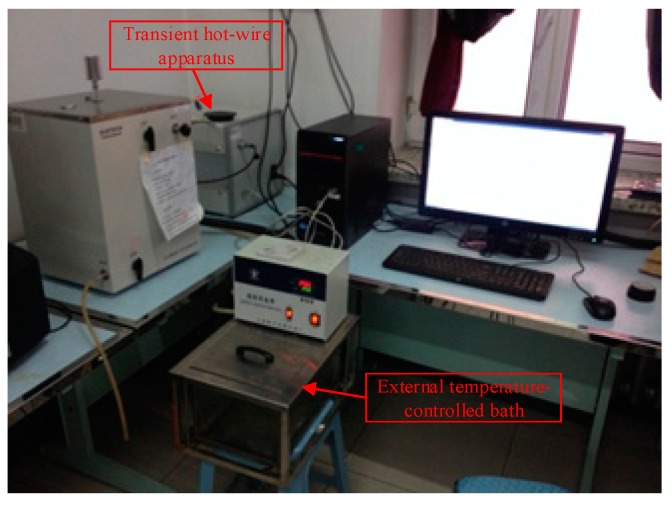
Measuring equipment of thermal conductivity for Al_2_O_3_-water nanofluids.

**Figure 4:**
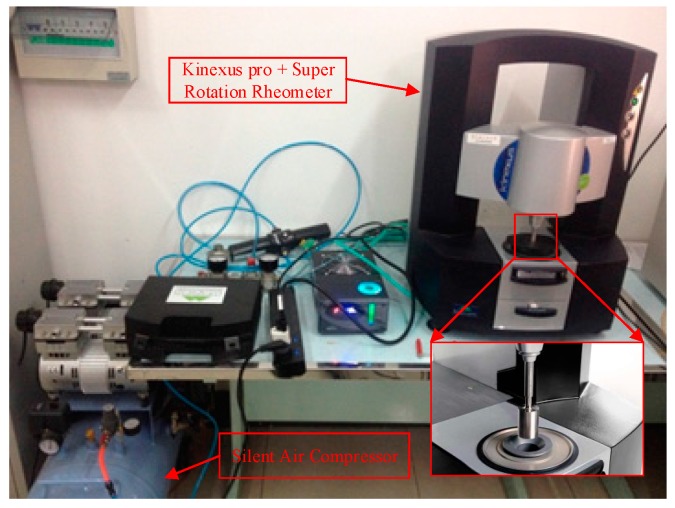
Measuring equipment of viscosity for Al_2_O_3_-water nanofluids.

To effectively measure the thermal conductivity of Al_2_O_3_-water nanofluids, a transient hot-wire apparatus designed by Xi’an Xiatech Electronic Technology Co., Ltd (Xi’an, China) is applied. The measuring accuracy of this apparatus is ±2%–3% in the thermal conductivity range of 0.001–20 W/m·K and temperature range of 113–423 K. Considering the constant temperature requirement, an external temperature-controlled bath is used, as shown in Figure 3. For the viscosity measurement of Al_2_O_3_-water nanofluids, the apparatus (Figure 4) including a Kinexus pro + Super Rotation Rheometer (Malvern Instruments Ltd, Malvern, UK) and a Silent Air Compressor (Shanghai Dynamic Industry Co., Ltd, Shanghai, China), is applied in the present experiment. Since the viscosities of Al_2_O_3_-water nanofluids are usually not very high, the Peltier Cylinder Cartridge is selected. The size of the cup is C14 (DIN standard). The diameter of the bob is 14 mm. Both the cup and bob are sandblasted to reduce slippage. The angular velocity of the rheometer is ranged from 10 nrad/s to 500 rad/s. The temperature resolution of this viscosity measuring equipment is 0.01 K in the temperature range of 233–473 K. More detailed devices information and the experimental procedure for viscosity measurement are referenced in [47,48,49]. 

## 3. Modeling Method Based on ANN

As an effective data-driven modeling approach, an ANN is put forward based on the inspiration from the human brain’s structure and activity mechanism. Nowadays, there are many different ANNs for various applications. In the fields of curve-fitting and nonlinear predictive modeling, the RBF neural network exhibits better ability in comparison with others [43].

### 3.1. RBF Neural Network Theory

**Figure 5:**
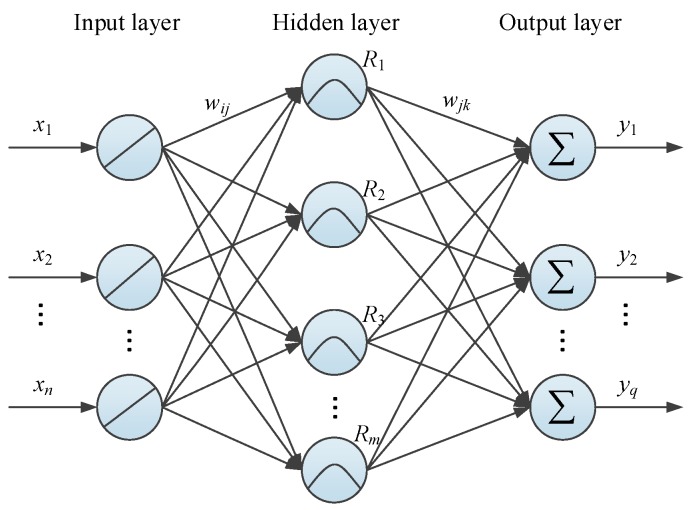
Typical architecture of an radial basis function (RBF) neural network.

In general, an RBF neural network (as shown in Figure 5) is constituted by an input layer, hidden layer, and output layer. The input and output layer correspond to the dendrite and synapse of biological neurons, respectively. Similarly to the function of cyton, the hidden layer plays a role of intermediation to process the input-output information and deliver it to the output layer. The connections between different layers are established through a series of artificial neurons and weights. Theoretically, the modeling process of an RBF neural network is to solve the mapping from $X^{n}$ to $Y^{q}$ (n,q≥1). Assuming the input vector of an RBF neural network is X, the response of kth neuron in the output layer ($y_{k}\in Y^{q}$) can be obtained using the following linear weighting function [50]:

(2) $$\begin{array}{cc} y_{k}=\sum_{j=1}^{m}\omega_{jk}R_{j}(X), & \left(k=1,2,\cdots ,q\right) \end{array}$$

where $\omega_{jk}$ is the connection weight between the jth hidden layer neuron and the kth output layer neuron and m and q are the numbers of neurons in the corresponding layer, respectively.

Different from those of many other ANN, the responses of RBF neural networks’ jth hidden layer neuron are usually determined by the RBF. When it selects the Gauss function, the corresponding $R_{j}(X)$ can be defined as:

(3) $$\begin{array}{cc} R_{j}(X)=\exp(-\frac{{\left\|X-c_{j}\right\|}^{2}}{2\sigma_{j}^{2}}), & \left(j=1,2,\cdots ,m\right) \end{array}$$

where ‖‖ is the Euclidean distance between input vector X and the jth neuron center $c_{j}$ and $\sigma_{j}$ is width of the jth neuron.

Analyzing Equations (2) and (3), it can be easily found that the key to RBF neural network training is how to determine $\omega_{jk}$, $c_{j}$, and $\sigma_{j}$. In the past decades, different unsupervised and supervised algorithms have been developed to solve the above problem [51]. In this study, the network parameters are updated by using a orthogonal least square (OLS) approach, for which the minimizing function is shown in Equation (4). More detailed information about OLSs can be found in [52].

(4) $$\min J=\sum_{k=1}^{q}\left({\left|y_{nk}-y_{dk}\right|}^{2}\right)$$

where $y_{nk}$ and $y_{dk}$ are the network output and desired output of the kth output layer node, respectively. 

### 3.2. Implementing Procedure

**Figure 6:**
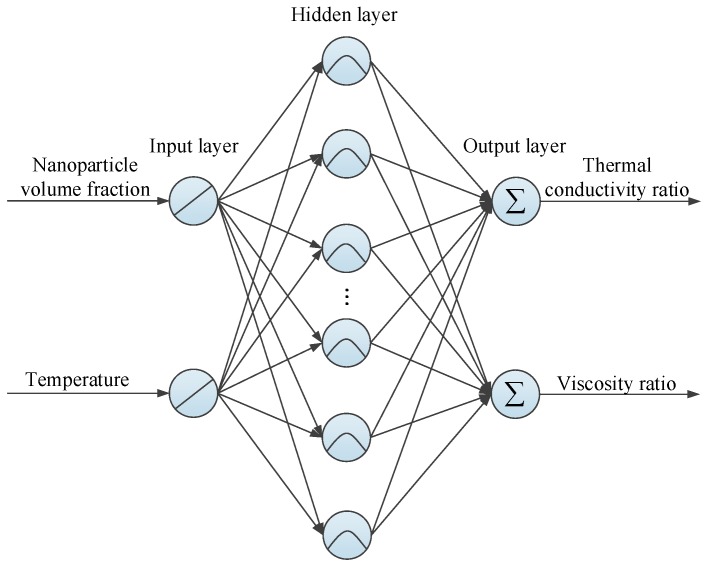
The RBF neural network developed in this study.

**Figure 7:**
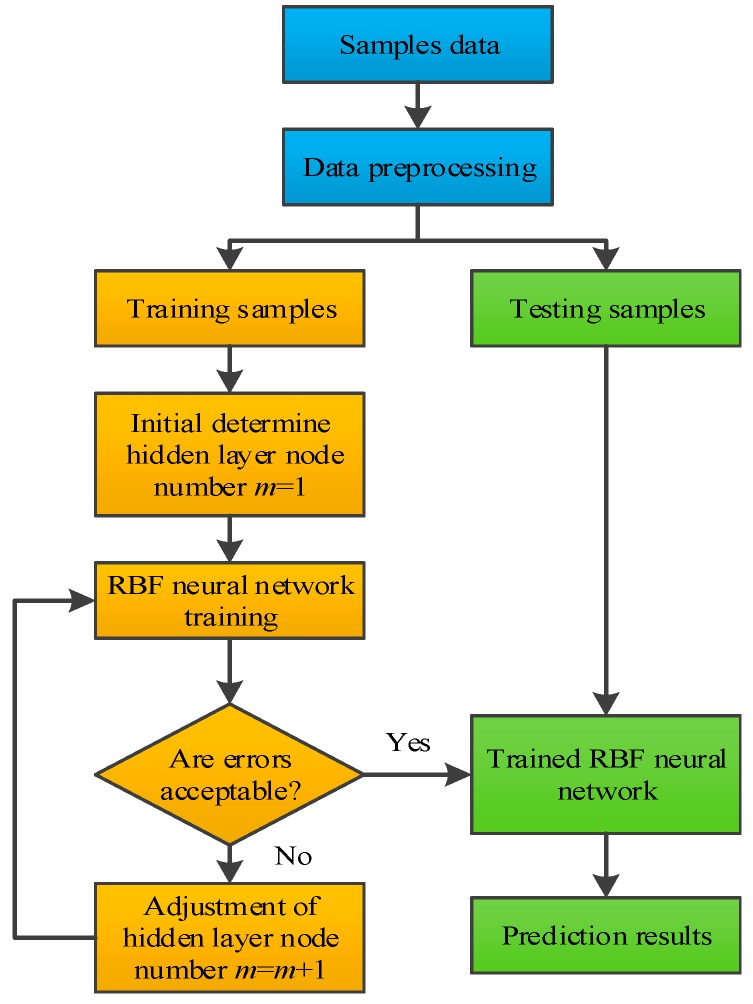
Implementing process of the RBF neural network for modeling and prediction.

In the present investigation, a typical three layer RBF neural network is developed. For the Al_2_O_3_-water nanofluids with the determined nanoparticle size, nanoparticle volume fraction and temperature are the most important factors for influencing the thermal conductivity and viscosity. Therefore, both the input and output layers of the RBF neural network consist of two neurons, as illustrated in Figure 6. The neurons in the hidden layer and others are determined in the training process. Figure 7 presents the detailed procedure for implementing the modelling and prediction of nanofluids based on the RBF neural network. To improve the training accuracy, all the input and output variables are normalized.

(5) $$x\prime =\frac{x-x_{\min}}{x_{\max}-x_{\min}}$$

where x is the original value, x′ is the normalized value, and $x_{\max}$ and $x_{\min}$ are the corresponding maximum and minimum of x.

To effectively evaluate the predictive accuracy of the RBF neural network, four important parameters, namely root mean squared error (*RMSE*), mean absolute percentage error (*MAPE*), sum of squared error (*SSE*), and statistical coefficient of multiple determination (*R*^2^), are used.

(6) $$RMSE={\left(\frac{1}{t}\sum_{l=1}^{t}{\left|P_{l}-Q_{l}\right|}^{2}\right)}^{1/2}$$

(7) $$MAPE=\frac{100\%}{t}\sum_{l=1}^{t}\left|\frac{P_{l}-Q_{l}}{P_{l}}\right|$$

(8) $$SSE=\sum_{l=1}^{t}{\left(P_{l}-Q_{l}\right)}^{2}$$

(9) $$R^{2}=1-\frac{\sum_{l=1}^{t}{\left(P_{l}-Q_{l}\right)}^{2}}{\sum_{l=1}^{t}{\left(P_{l}\right)}^{2}}$$

where *P* is the desired value, *Q* is the network output value, and *t* is the number of samples.

## 4. Results and Discussion

### 4.1. Enhancement of Thermal Conductivity

To verify the effectiveness of the above thermal conductivity measuring apparatus, water is measured first. Considering the temperature balance of the testing sample and the fluid in bath, the testing temperature can be determined when it remains constant for 20 min. Every experimental data is the average value of five measurements with a frequency interval of 5 min. Table 1 presents the experimental thermal conductivity of water in the temperature range of 288–318 K. According to the comparison, it is concluded that the thermal conductivity apparatus has good precision for the present study. Based on the experimental data, the measurement uncertainty of thermal conductivity is less than 5% for water.

**Figure 8:**
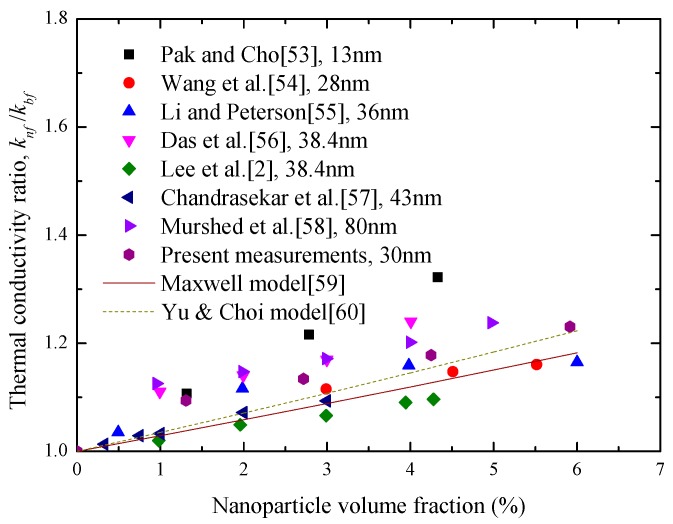
Variations of Al_2_O_3_-water nanofluids $k_{nf}/k_{bf}$ with nanoparticle volume fraction at room temperature [2,53,54,55,56,57,58,59,60].

Figure 8 presents the change of the thermal conductivity ratio ($k_{nf}/k_{bf}$) between Al_2_O_3_-water nanofluids and water with different nanoparticle volume fractions at room temperature. From Figure 8, it is found that the thermal conductivity of water can be enhanced obviously with the increase of the Al_2_O_3_ nanoparticle. For example, at a nanoparticle volume fraction of 1.31%, the enhancement of water thermal conductivity is 9.4%. When the volume fraction of the Al_2_O_3_ nanoparticle increases to 5.92%, the $k_{nf}/k_{bf}$ can change to 1.231. In addition, Figure 8 compares the present measurements with many experimental data obtained from the existing publications. The results show that they are in good agreement with both the qualitative and quantitative aspects. This may mean that both the sample preparation and thermal conductivity measurements are successful. In addition, it is also clearly observed from Figure 8 that the enhancement of the Al_2_O_3_ nanoparticle on water thermal conductivity cannot be described accurately by using the well-known Maxwell model and the Yu and Choi model due to the complex influence mechanisms such as the interfacial layer, nanoparticle Brownian motion, and clustering.

Maxwell model [59]

(10) $$\frac{k_{nf}}{k_{bf}}=\frac{k_{p}+2k_{bf}+2\varphi_{p}(k_{p}-k_{bf})}{k_{p}+2k_{bf}-\varphi_{p}(k_{p}-k_{bf})}$$

where $k_{nf}$, $k_{p}$, and $k_{bf}$ are the thermal conductivity of nanofluids, the nanoparticle, and base fluid, respectively.

Yu & Choi model [60]

(11) $$\frac{k_{nf}}{k_{bf}}=\frac{k_{pl}+2k_{bf}+2(k_{pl}-k_{bf}){\left(1+\beta \right)}^{3}\varphi_{p}}{k_{pl}+2k_{bf}-(k_{pl}-k_{bf}){\left(1+\beta \right)}^{3}\varphi_{p}}$$

(12) $$k_{pl}=\frac{[2(1-\gamma )+{\left(1+\beta \right)}^{3}(1+2\gamma )]\gamma }{-(1-\gamma )+{\left(1+\beta \right)}^{3}(1+2\gamma )}k_{p}$$

where $\gamma =k_{l}/k_{p}$, $k_{l}$ is the thermal conductivity of interfacial layer, $\beta =h/r_{p}$, h is thickness of interfacial layer, and $r_{p}$ is the radius of nanoparticle.

**Figure 9:**
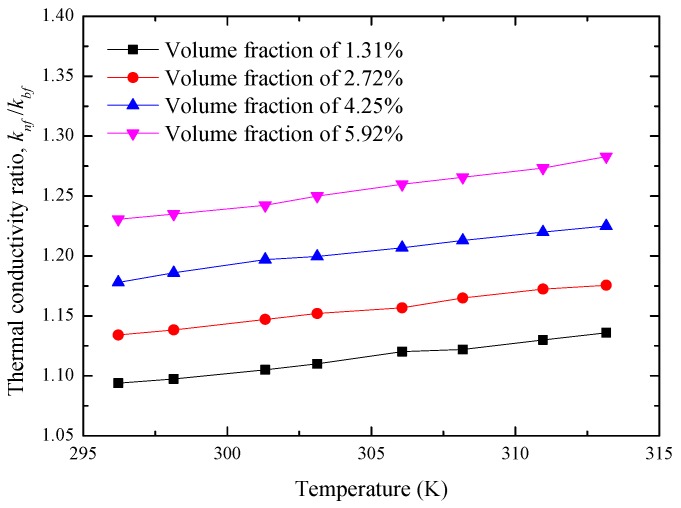
Variations of $k_{nf}/k_{bf}$ with temperature for different nanoparticle volume fractions of Al_2_O_3_-water nanofluids.

Considering the effects of temperature ranging from 296 to 313 K, Figure 9 presents the variation of $k_{nf}/k_{bf}$ with various volume fractions. It can be found that, for any volume fraction of the Al_2_O_3_ nanoparticle, the corresponding $k_{nf}/k_{bf}$ can linearly improve with the increase of temperature, which is usually explained by the enhancement of nanoparticle Brownian motion. In the present study, taking the nanofluids with an Al_2_O_3_ volume fraction of 2.72% as an example, the maximum $k_{nf}/k_{bf}$ of 1.283 is obtained at the nanoparticle volume fraction of 5.92% and a temperature of 313 K.

### 4.2. Viscosity Investigation

Before experimentally analyzing the viscosity of Al_2_O_3_-water nanofluids, it is also necessary to evaluate the apparatus’s effectiveness by selecting water as a sample. Both the measuring frequency and data analysis method are same as those for thermal conductivity. From the contrastive analysis shown in Table 2, it can be inferred that the measurements of viscosity are effective, with a maximum deviation of 0.988% in the temperature ranges of 288–318 K. In addition, the experimental analysis indicates that the measurement uncertainty of water viscosity is less than 5% using the above mentioned approach when the shear rate changes.

**Figure 10:**
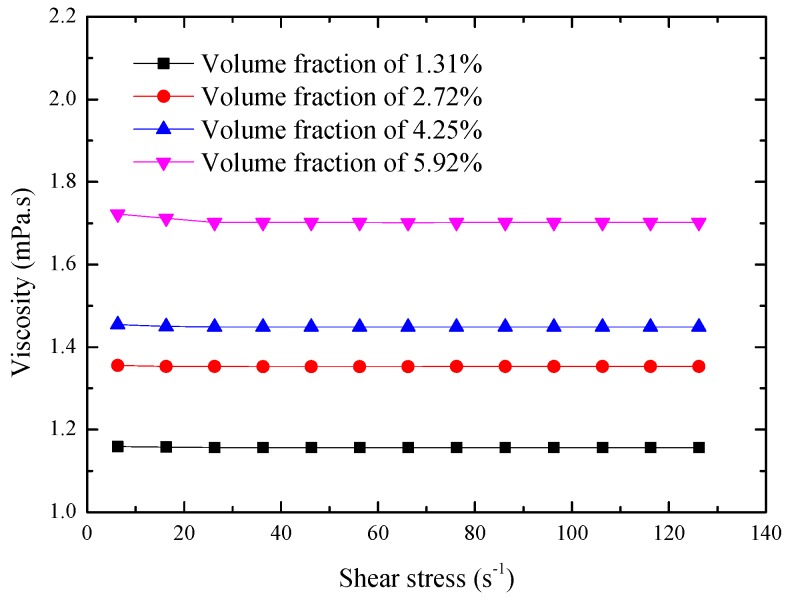
Rheological behaviors of Al_2_O_3_-water nanofluids at room temperature.

To investigate the influence of Al_2_O_3_-water nanofluids, Figure 10 presents the relationship between the shear rate and nanofluid viscosity at the temperature of 298 K. The results show that, with the increase of the shear rate from 6.326 s^−1^ to 126.2 s^−1^, the viscosities of Al_2_O_3_-water nanofluids with different Al_2_O_3_ volume fractions do not change significantly. This may mean that the viscosities of the Al_2_O_3_-water nanofluids obtained in the present study display Newtonian behavior. 

**Figure 11:**
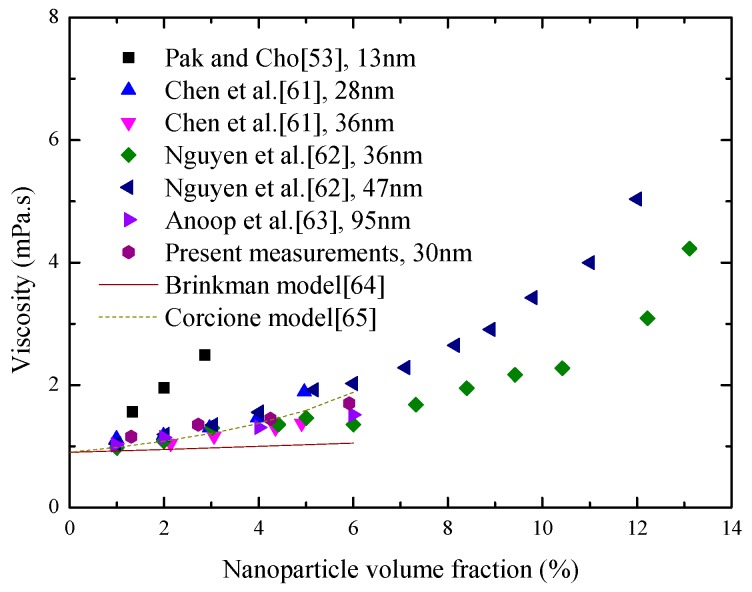
Variations of Al_2_O_3_-water nanofluids’ viscosity with nanoparticle volume fraction at room temperature [53,61,62,63,64,65].

Considering the influence of the Al_2_O_3_ volume fraction at the temperature of 298 K, the experimental viscosities of the Al_2_O_3_-water nanofluids are given and compared with much published data in Figure 11. All the results show that the suspension of Al_2_O_3_ nanoparticles can increase the viscosity of water, and there is a slight non-linear relationship between the viscosity of nanofluids and nanoparticle volume fraction. Moreover, a careful inspection of Figure 11 reveals that the theoretical viscosities obtained by the classical Brinkman model are significantly lower than the corresponding measurements. Compared to the Brinkman model, the Corcione model can effectively describe the effect of nanoparticle volume fraction on viscosity, but its prediction precision is not very ideal. This is because the viscosity of nanofluids depends strongly on many known and unknown factors.

The Brinkman model [64] is as follows:

(13) $$\frac{\mu_{nf}}{\mu_{bf}}=\frac{1}{{\left(1-\varphi_{p}\right)}^{2.5}}$$

where $\mu_{nf}$ and $\mu_{bf}$ are the viscosity of nanofluids and base fluid, respectively.

The Corcione model [65] is as follows:

(14) $$\frac{\mu_{nf}}{\mu_{bf}}=\frac{1}{1-34.87{\left(d_{p}/d_{f}\right)}^{-0.3}{\varphi_{p}}^{1.03}}$$

(15) $$d_{f}={\left[\frac{6M}{N\pi \rho_{f0}}\right]}^{1/3}$$

where M is the molar mass of base fluid molecule, $N=6.022\times 10^{23}mol^{-1}$ is avogadro’s number, $\rho_{f0}$ is the density of base fluids at temperature of 293 K, and $d_{p}$ is the diameter of nanoparticle.

**Figure 12:**
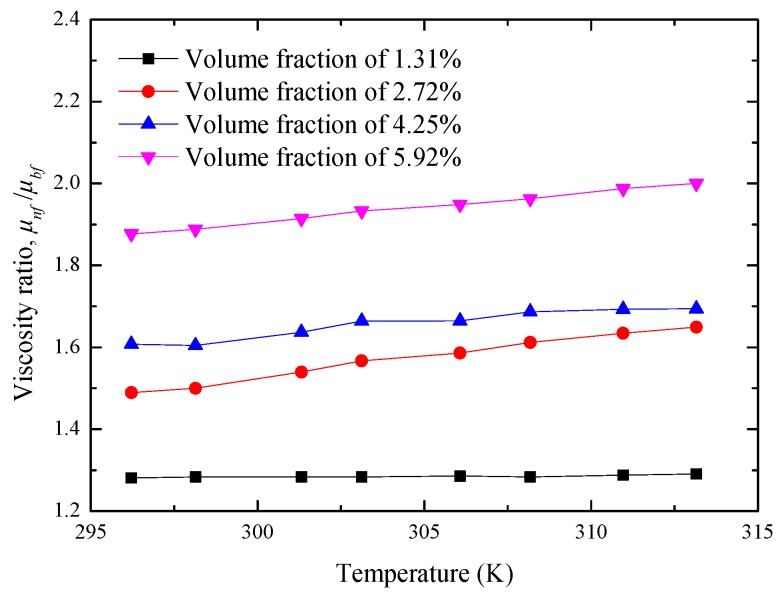
Variations of $\mu_{nf}/\mu_{bf}$ with temperature for different nanoparticle volume fractions of Al_2_O_3_-water nanofluids.

Figure 12 presents the variation of the viscosity ratio, $\mu_{nf}/\mu_{bf}$, between nanofluids and water as the functions of temperature and nanoparticle volume fraction. From Figure 12, it is observed that, for the manufactured Al_2_O_3_-water nanofluids in this study, temperature has an enhanced effect on viscosity in the temperature ranges of 296–313 K. At the nanoparticle volume fraction of 4.25%, the $\mu_{nf}/\mu_{bf}$ fractions are respectively 1.605, 1.664, 1.687, and 1.694 when the temperatures are 298 K, 303 K, 308 K, and 313 K.

### 4.3. Predictive Analysis of RBF Neural Networks

Based on the above experiment, the limited experimental data (40) are used to discuss the modeling and prediction processes of the RBF neural network for the thermal conductivity and viscosity of Al_2_O_3_-water nanofluids. Among them, the ratio of training and testing samples is 3:1.

**Figure 13:**
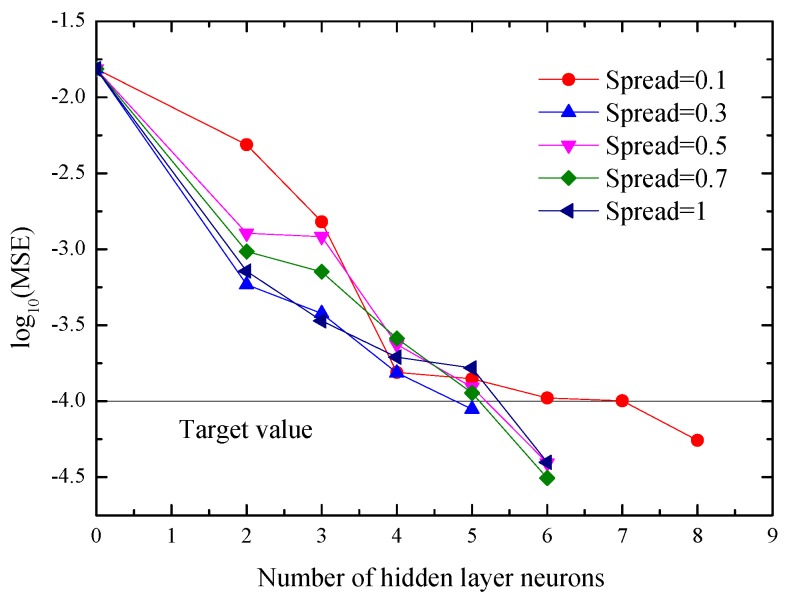
Relationships of mean square error (MSE) and the number of hidden layer neurons with different values of Spread.

For the RBF neural network, Spread is usually a very important factor for influencing the training process. Figure 13 shows the relationships of mean square error (MSE) and the number of hidden layer neurons with different values of Spread. Table 3 lists the effect of Spread on network modeling accuracy. Comprehensively analyzing the results reported in Figure 13 and Table 3, it is found that both the network structure and modeling performance cannot be changed significantly with different values of Spread in this study. Therefore, the network structure of 2-8-2 neurons with the Spread of 0.1 is used. The related weights and biases of a 2-8-2 RBF neural network can be found in Table 4. 

**Figure 14:**
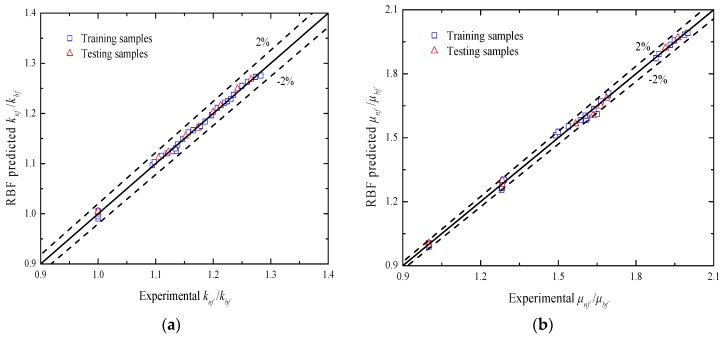
Scatter plots of (**a**) $k_{nf}/k_{bf}$ and (**b**) $\mu_{nf}/\mu_{bf}$ for the RBF predicted results and experimental data.

**Figure 15:**
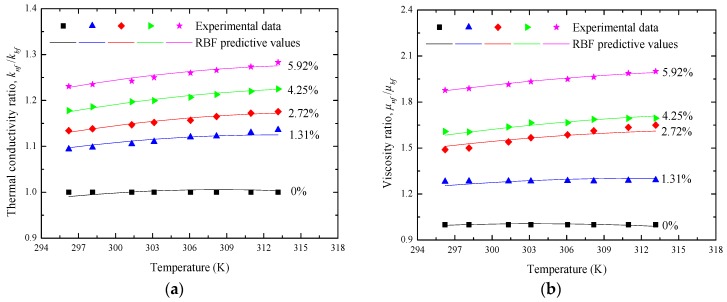
Comparisons of the experimental and RBF predicted results for (**a**) $k_{nf}/k_{bf}$ and (**b**) $\mu_{nf}/\mu_{bf}$.

Figure 14 and Figure 15 compare the RBF predicted thermophysical properties of Al_2_O_3_-water nanofluids with the corresponding experimental data. Table 5 lists the predictive evaluation criteria of the RBF neural network for the training and testing samples. As shown in Figure 14 and Table 5, the RBF neural network has a high accuracy for modeling the thermal conductivity and viscosity of Al_2_O_3_-water nanofluids with limited experimental data. All the prediction errors of thermal conductivity and nearly 92.5% of those of viscosity are within the ±2% error band. It is worth noting that there is a higher accuracy for the testing dataset but not the training ones. This is because the samples of the testing dataset are very few in this study. In addition, the results analysis of Figure 15 reveals that the effects of nanoparticle volume fraction and temperature on the above two thermophysical properties can be effectively extracted in the data-driven prediction of the RBF neural network. All of the above investigations demonstrate that a RBF neural network provides a successful alternative to the traditional model-based prediction approach for the thermal conductivity and viscosity of Al_2_O_3_-water nanofluids.

## 5. Conclusions

In this paper, the experiments on Al_2_O_3_-water nanofluid preparation and thermophysical properties measurement are performed to obtain the effects of nanoparticle volume fraction and temperature on thermal conductivity and viscosity. All the experimental results showed that both thermal conductivity and viscosity could be enhanced with the increase of the Al_2_O_3_ nanoparticle volume fraction and temperature. On this basis, considering the advantage of a RBF neural network in modeling, a case study was investigated to discuss the application of a RBF neural network on the prediction of nanofluids’ thermal conductivity and viscosity with 40 sets of experimental data. By comparing the RBF predictive values and the experimental data, it was demonstrated that RBF neural network not only exhibited good modeling accuracy (thermal conductivity: RMSE = 8.572 × 10^−3^, MAPE = 0.5177%, SSE = 2.939 × 10^−3^, $R^{2}$ = 0.999944; viscosity: RMSE = 1.423 × 10^−2^, MAPE = 0.5618%, SSE = 8.094 × 10^−3^, $R^{2}$ = 0.999913), but also could effectively extract the influences of nanoparticle volume fraction and temperature on Al_2_O_3_-water nanofluids’ thermal conductivity and viscosity. 

## Figures and Tables

**Table 1:** Thermal conductivity measurement and analysis for water.

Temperature (K)	Reference Values (W/(m·K))	Measure Values (W/(m·K))	Deviation (%)
288	0.5916	0.595	0.5747
293	0.6003	0.6019	0.2665
298	0.6088	0.6111	0.3778
303	0.6173	0.6185	0.1944
308	0.6245	0.6252	0.1121
313	0.6318	0.6292	−0.4115
318	0.6379	0.6374	−0.0784

**Table 2:** Viscosity measurement and analysis for water.

Temperature (K)	Reference Values (mPa·s)	Measure Values (mPa·s)	Deviation (%)
288	1.1426	1.1517	−0.7955
293	1.0094	0.9998	0.9517
298	0.8938	0.8943	−0.0658
303	0.8029	0.7958	0.8791
308	0.7226	0.7182	0.6106
313	0.6634	0.6693	−0.8866
318	0.6008	0.6043	−0.5876

**Table 3:** Performance evaluation of the RBF neural network for the total samples with different values of Spread.

Parameters	Evaluation Criteria	Spread				
		0.1	0.3	0.5	0.7	1
*k_nf_*/*k_bf_*	*RMSE*	8.572 × 10^−3^	6.797 × 10^−3^	4.140 × 10^−3^	4.346 × 10^−3^	4.043 × 10^−3^
	*MAPE*(%)	0.5177	0.4803	0.2872	0.3197	0.2866
	*SSE*	2.939 × 10^−3^	1.848 × 10^−3^	6.857 × 10^−4^	7.556 × 10^−4^	6.538 × 10^−4^
	*R*^2^	0.999944	0.999965	0.999987	0.999986	0.999988
*μ_nf_*/*μ_bf_*	*RMSE*	1.423 × 10^−2^	2.311 × 10^−2^	1.624 × 10^−2^	1.381 × 10^−2^	1.658 ×10^−2^
	*MAPE*(%)	0.5618	1.3862	0.8233	0.7169	0.8280
	*SSE*	8.094 × 10^−3^	2.137 × 10^−2^	1.055 × 10^−2^	7.634 × 10^−3^	1.100 × 10^−2^
	*R*^2^	0.999913	0.999770	0.999887	0.999918	0.999882

**Table 4:** Weight and bias coefficients of the developed RBF neural network.

Neuron	Hidden Layer			Output Layer		
	Weights *w_ij_* and Biases			Weights *w_jk_* and Biases		
	Nanoparticle Volume Fraction	Temperature	Biases	Thermal Conductivity	Viscosity	Biases
1	0.7184	1	1.1894	−61.8011	−143.4012	−4.7283
2	0	1	1.1894	−4.4132	−18.1112	−6.1187
3	1	1	1.1894	24.4808	49.7229	
4	0.4595	1	1.1894	92.2675	218.6915	
5	0.2208	0.9930	1.1894	−76.4725	−178.5244	
6	0	0.9841	1.1894	36.6988	86.1722	
7	0.7184	1	1.1894	−61.8011	−143.4012	
8	0	1	1.1894	−4.4132	−18.1112	

**Table 5:** Performance evaluation of the RBF neural network for the training and testing samples.

Parameters	Evaluation Criteria	Training Samples	Testing Samples
*k_nf_*/*k_bf_*	*RMSE*	4.464 × 10^−3^	3.974 × 10^−3^
	*MAPE* (%)	0.3230	0.3098
	*SSE*	5.977 × 10^−4^	1.579 × 10^−4^
	*R*^2^	0.999985	0.999988
*μ_nf_*/*μ_bf_*	*RMSE*	1.419 × 10^−2^	1.263 × 10^−2^
	*MAPE* (%)	0.7472	0.6261
	*SSE*	6.040 × 10^−3^	1.594 × 10^−3^
	*R*^2^	0.999913	0.999932
